# Genetic variants associated with gestational diabetes mellitus: a meta-analysis and subgroup analysis

**DOI:** 10.1038/srep30539

**Published:** 2016-07-29

**Authors:** Ling Wu, Long Cui, Wing Hung Tam, Ronald C. W. Ma, Chi Chiu Wang

**Affiliations:** 1 Department of Obstetrics & Gynaecology, The Chinese University of Hong Kong, Hong Kong; 2 Department of Medicine and Therapeutics, The Chinese University of Hong Kong, Hong Kong; 3 Hong Kong Institute of Diabetes and Obesity, The Chinese University of Hong Kong, Hong Kong; 4 Li Ka Shing Institute of Health Sciences, The Chinese University of Hong Kong, Hong Kong; 5 School of Biomedical Sciences, The Chinese University of Hong Kong, Hong Kong

## Abstract

Previous studies have demonstrated that gestational diabetes mellitus (GDM) and Type 2 diabetes mellitus (T2D) share common genetic polymorphisms. We conducted meta-analysis and subgroup analysis of all available variants and determined the effects of confounding and experimental components on the genetic association of GDM. Any case-controlled or cohort studies with genotype distribution compared GDM cases with controls were included. In total, 28 articles including 8,204 cases and 15,221 controls for 6 polymorphisms were studied. rs10830963(*MTNR1B*), rs7903146(*TCF7L2*), and rs1801278(*IRS1*) were significantly associated with the increased GDM risk. The association of rs4402960(*IGF2BP2*) and rs1800629(*TNF-α*) was significant only when the studies with control allele frequency deviation and publication bias were excluded. Further subgroup analysis showed the risk alleles of rs7903146(*TCF7L2*) and rs1801282(*PPARG*) were significantly associated with the GDM risk only in Asian, but not in Caucasian population. The OGTT test using 100 g, but not 75 g; and genotype detection by other assays, but not Taqman method, were also significantly associated with increased GDM risk in rs1801278(*IRS1*) and rs7903146(*TCF7L2*). Overall GDM was associated with rs10830963(*MTNR1B*), rs7903146(*TCF7L2*), and rs1801278(*IRS1*), but only rs7903146(*TCF7L2*) and rs1801282(*PPARG*) were significant in Asian populations. While rs1801278(*IRS1*) and rs7903146(*TCF7L2*) were significantly affected by OGTT protocol and genotyping methods.

Gestational diabetes mellitus (GDM), one of the most common pregnancy complications, is defined as the onset or first recognition of glucose intolerance during pregnancy^1^. Adverse pregnancy outcomes of GDM impact on both mothers and their offspring during and after pregnancy^2^. Pregnant women complicated with GDM tend to have increased risk of miscarriage, hypertensive disorders, macrosomia, operative delivery and postpartum hemorrhage; and 2.6% to 70% will develop diabetes mellitus 28 years later^3^. The offspring of these women are associated with large for gestational age, premature birth, neonatal respiratory distress syndrome, hypoglycemia, and also impaired glucose metabolism in early age^4^.

Globally, the prevalence of GDM varies in population and ethnicity from 1% to 14%^7^. GDM affects 5% to 6% of all pregnancies in the U.S.A.^5^; <5% in South Korea, South Africa and United Kingdom; <10% in Italy and Australia; and nearly 20% in Bermuda and Nepal^4^. GDM received increasing attention globally due to its continuous increase in prevalence, particular in developing countries, including China, India and Africa^6^. For example, the prevalence of GDM was increased from 2.3% in 1999 to 6.8% in 2008 in northern China^7^.

GDM and Type 2 diabetes mellitus (T2D) have similar pathogenesis with impaired insulin secretion and increased insulin resistance^8^. Previous studies demonstrated that GDM and T2D share common genetic polymorphisms, with similar magnitude of the effect sizes on the same risk alleles^9^. Recent meta-analysis demonstrated association of individual genetic variants with the risk of GDM^10^. However, most analyses were limited by only examining single or small numbers of genetic variants, thus sample sizes were not sufficient^11^. The interaction between genetic polymorphisms and other confounding risk factors; and the effects of study populations, demographic characteristics and detection methods were commonly neglected^12^,^13^. In this study, we conducted a large scale meta-analysis and further subgroup analysis of most GDM associated genetic variants whose pathogenesis is similar to those of T2D in order to confirm its genetic association and dissect the effects of ethnicity, sample size, OGTT criteria, maternal age, parity, BMI and genotyping methods on the association.

## Methods

### Literature search

All recent genetic association studies published in recent years (2004 to 2015) were searched in Medline, Evidence Based Medicine (EBM), Pubmed and Web of Science (ISI) for studies published in English and in the WanFang and China National Knowledge Infrastructure (CNKI) database for literature published in Chinese. Keywords in the searches included “gestational diabetes mellitus”, “GDM”, and “genetic”. In addition, the references cited by the original studies, reference lists, conference proceedings, and Google scholar were also searched. Articles published in language other than English and Chinese were not included in this analysis.

### Eligibility criteria

Inclusion criteria included any case-controlled or cohort studies with genotype distribution information in both GDM cases and controls. Exclusion criteria included review papers, commentary articles, publication in other languages, studies of postpartum diabetes mellitus, duplicated reports and case series. Genetic studies with no information on genetic polymorphisms, incomplete genotyping data and family trio studies were also excluded. For meta-analysis, a genetic variant reported by less than 3 independent studies and meta-analysis papers were further excluded.

### Study selection and validity assessment

Data extraction was independently completed by two reviewers (L.W. and L.C.) from all eligible publications. If a consensus could not be reached, a third reviewer (C.C.W.) settled the discrepancy. If any outstanding queries remain, the corresponding authors of the publication were contacted for data clarification. When the data were not available in the published articles or a further clarification was needed, we contacted the authors by e-mail.

### Data extraction and Statistical analysis

Studied variants and its molecular functions in T2D/GDM were recorded. Information was extracted from each selected study, including names of the authors, publication year, study ethnicity, sample size and the definition of cases and controls, the diagnostic criterion for GDM used in the study, the mean age and BMI of cases and controls, genotyping methods, and the frequency of risk alleles were defined according to genotypes. Pooled odds ratio (OR) and 95% confidence intervals (CI) were calculated by allele models of the selected studies on the same variant. Significance levels were determined by Z test. Forest plots were used to demonstrate effect sizes and their confidence intervals (CI)^14^. Heterogeneity amongst the studies of the genetic variants was assessed by *I*^*2*^ statistic which reflected the heterogeneity proportion in the total variation of effect size. If I^2^ > 50%, random-effects model (REM) was used. While I^2^ ≤ 50%, fixed-effects model (FEM) was used to determine the significant heterogeneity^15^. Hardy-Weinberg equilibrium (HWE) was tested to assess the stability of allele and genotype frequencies in the study population of each study. χ2 test by Fisher’s exact method was used to estimate whether there was significant deviation from HWE among the control group. Significant association was repeatedly determined by either no, HWE only, or combined HWE and Funnel outlier exclusion. All statistical analyses were carried out using Stata software version 12.1 (Stata Corporation, College Station, TX, USA). The significance level was set as 0.05, except Cochran’s Q test for heterogeneity as 0.1.

### Subgroup analysis

Effects of different confounding factors of GDM, demographic characteristics of the study populations, diagnostic criteria of GDM, and study methodology of the genetic association studies were tested by subgroup analysis. For the confounding factors, maternal age (<35 vs ≥35), previous history of GDM (yes vs. no) and BMI before and during pregnancy (<25 vs ≥25) were compared. For demographic data, parity (nullpara vs multipara) and gestational age (2^nd^ trimester vs 3^rd^ trimester) were compared. For the diagnosis of GDM, diagnostic criteria using different approaches (75 g OGTT vs 100 g OGTT) were compared. For study methods, genotyping methods (Taqman vs others), study ethnicity (Asian vs Caucasian) and sample sizes (small vs large) were also compared. Sample size was defined as small or large according to calculation using an online sample size estimator (OSSE) on the included papers for each variant (Supplementary Table 1). The confounding and experimental factors of a genetic variant in less than 3 independent studies were excluded due to insufficient data for subgroup analysis. Pooled odds ratios (ORs) and 95% confidence intervals (CI) in each subgroup were calculated as above. χ^2^ test by Cochran Q model was used to detect the heterogeneity between subgroups.

### Publication bias of the included studies

Funnel plots were employed to check the potential publication bias due to significant deviation in the CI distribution from the other studies^16^. Egger’s test was used to assess the possibility of publication bias by outlier distribution of the standard error of log OR.

### Sensitivity analysis of the meta-analysis

To test the robustness of uncertainty in the meta-analysis, sensitivity analyses were employed.

## Results

### Characteristics of the included studies

**Figure 1:**
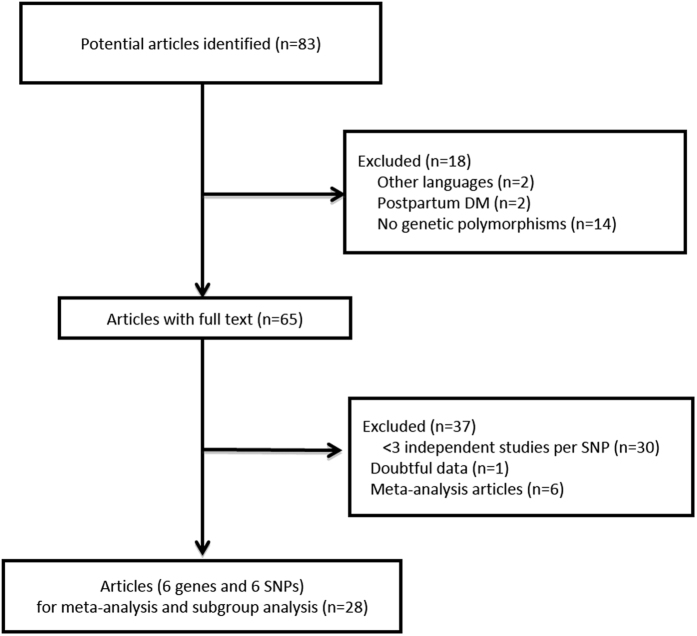
Flowchart of the systematic search methodology.

Literature search identified 83 potential articles, including 75 from PubMed and ISI (2004–2015) and 8 from the WanFang database (2004–2015). After abstracts were screened, 18 articles were excluded: 2 published in other languages (Polish and Czech), 2 studies which included postpartum DM, 14 which had no information on genetic polymorphisms. In addition, 37 articles were excluded for the different reasons as listed: 30 articles whereby the reported genetic variants have been studied in less than 3 independent studies for appropriate meta-analysis, 6 which were published meta-analysis and 1 which had doubtful data with potential reversed wild-type allele numbers and risk allele numbers but could not be verified by contacting the author^17^. One study^18^ included for its control subjects both men and women with average age more than 60-year-old and no evidence of DM. In addition the frequency of polymorphism associated with DM is low, therefore this study was regarded as eligible for inclusion. In total, 28 articles (7 papers from China, 8 papers from other regions of Asia and 13 papers from Western countries) were included for the meta-analysis and subgroup-analysis. All included studies were case-controlled studies, including 8,204 cases and 15,221 controls for 6 loci (Fig. 1). Of the 6 loci, Insulin-like growth factor 2 mRNA-binding protein 2 (*IGF2BP2*), Melatonin receptor 1B (*MTNR1B*), Transcription factor 7-like 2 (*TCF7L2*) are involved in insulin secretion^19^,^20^,^21^; Insulin receptor substrate 1 (*IRS1*) and Peroxisome proliferator-activated receptor gamma (*PPARG*) are involved in insulin resistance^22^,^23^; while Tumor necrosis factor alpha (*TNF-α*) is involved in inflammation in DM and/or GDM pathogenesis (Supplementary Table 2)^24^. Though three variants (rs4402960 in *IGF2BP2*, rs10830963 in *MTNR1B* and rs7903146 in *TCF7L2*) are located at intron regions, these causal variants do modulate gene function and impact on downstream transcriptional machinery^25^. Another three variants (rs1801278 in *IRS1*, rs1801282 in *PPARG* and rs1800629 in *TNF-α*) are located at exon regions. Study characteristics, detailed genotype information and allele distribution in each included study are summarized in Supplementary Tables 3 and 4, respectively. Results of meta-analysis and sub-group analysis in each SNP are detailed as below. Sensitivity analysis did not identify any significant uncertainty in the analysis.

### Meta-analysis

#### Variants involved in insulin secretion

*IGF2BP2* (*rs4402960*) There were 3 articles which studied the T allele of rs4402960 but meta-analysis did not identify any significant association of the variant with the increased risk of GDM (pooled OR 1.12, 95% CI 0.89–1.40, P = 0.353, Table 1 and Supplementary Figure 1). There was significant heterogeneity across the studies (I^2^ = 68.4%; p = 0.042). There was no significant HWE variation (Supplementary Table 4), but there was potential publication bias (Supplementary Figure 3). After the outlier^19^ from the funnel plot analysis was removed, the association between the variant and GDM was significant (pooled OR 1.22, 95% CI 1.09–1.36; p < 0.001, Table 1 and Supplementary Figure 3) and heterogeneity was not significant (I^2^ = 0%; p = 0.637). There were not enough studies for subgroup analysis.

*MTNR1B* (*rs10830963*) There were 7 eligible studies which examined the rs10830963 polymorphism. Meta-analysis showed that the G allele was significantly associated with increased risk of GDM (pooled OR 1.31, 95% CI 1.18–1.47, p < 0.001; Heterogeneity I^2^ 44.2%, p = 0.097; Table 1, Supplementary Figure 1). There was significant departure from HWE in 2 studies^26^,^27^ (Supplementary Table 4) and the significant association was maintained when these studies were excluded (pooled OR 1.28, 95% CI 1.14–1.44, p < 0.001; Heterogeneity I^2^ 50.3%, p = 0.09; Table 1, Supplementary Figure 2). There was no publication bias under the funnel plot analysis (Supplementary Figure 3). There were enough studies for subgroup analysis for ethnicity, OGTT criteria, genotyping methods and sample size only. Studies looking at ethnicity found the risk allele was significantly associated with increased risk of GDM both in Asian populations (pooled OR 1.23, 95% CI 1.10–1.38, p < 0.001; I^2^ 37.7%, p = 0.186) and Caucasian populations (pooled OR 1.49, 95% CI 1.22–1.82, p < 0.001, Table 2). Studies either used 75 g or 100 g in OGTT for GDM (pooled OR 1.38, 95% CI 1.20–1.57, p < 0.001 and pooled OR 1.20, 95% CI 1.01–1.44, p = 0.043, respectively; Table 2); studies used Taqman assay or other assays as genotype detection method (pooled OR 1.28, 95% CI 1.08–1.51, p = 0.004 and pooled OR 1.29, 95% CI 1.08–1.54, p = 0.005, respectively; Table 2); and studies with both larger sample size (≥336) and smaller sample size (<336) (pooled OR 1.27, 95% CI 1.12–1.44, p < 0.001 and pooled OR 1.53, 95% CI 1.01–2.32, p = 0.047, respectively; Table 2) were significantly associated between the risk variant and GDM. But only studies included subjects with high mean pre-pregnancy BMI (≥25), but not low mean pre-pregnancy BMI (<25), were significantly associated between the risk variant and GDM (pooled OR 1.24, 95% CI 1.02–1.51, p = 0.033, Table 2).

*TCF7L2* (*rs7903146*) There were 9 published articles which studied the rs7903146 polymorphism. Meta-analysis showed that the T allele was significantly associated with increased risk of GDM (pooled OR 1.41, 95% CI 1.16–1.72, p = 0.001; Heterogeneity I^2^ 80.8%, p < 0.001; Table 1 and Supplementary Figure 1). HWE was significant in 2 studies^28^,^29^ (Supplementary Table 4), the significant association was maintained when those studies were removed (pooled OR 1.40, 95% CI 1.03–1.90, p = 0.032; Heterogeneity I^2^ 85.1%, p < 0.001 Table 1 and Supplementary Figure 2). Funnel plot analysis indicated potential publication bias in 1 study^30^ (Supplementary Figure 3), the association was still significant when this study was excluded (pooled OR 1.57, 95% CI 1.38–1.79, p < 0.001; Heterogeneity I^2^ 9%, p = 0.359; Table 1 and Supplementary Figure 4). Only ethnicity, OGTT criteria and genotyping methods have enough studies for further subgroup analysis. Comparing studies in different ethnicity showed the risk allele was significantly associated with the increased risk of GDM in the Asian population only (pooled OR 1.58, 95% CI 1.12–2.23, p = 0.009; I^2^ 39.1%, p = 0.194), but not in Caucasian population (pooled OR 1.32, 95% CI 0.86–2.03, p = 0.212; I^2^ 91.4%, p = 0.212); Table 2). Studies which either used 75 g or 100 g in OGTT for GDM diagnosis had no statistical significant association between the risk alleles and GDM (Table 2). If studies with potential publication bias were not included in subgroup analysis, the association became significant in Caucasian population (pooled OR 1.55, 95% CI 1.35–1.79, p < 0.001; heterogeneity I^2^ 6.3%, p = 0.344) and GDM diagnosis using 75 g OGTT (pooled OR 1.62, 95% CI 1.39–1.89, heterogeneity I^2^ 19.8%, p = 0.291) only (Supplementary Table 5). Studies which used methods other than Taqman assay as genotype detection were also significant (Table 2).

### Variants involved in insulin resistance

*IRS1* (*rs1801278*) Five eligible articles studied rs1801278 polymorphism. Meta-analysis showed that the T allele was significantly associated with the increased risk of GDM (pooled OR 1.53, 95% CI 1.08–2.15, p = 0.015; Heterogeneity I^2^ 49.6%, p = 0.094; Table 1 and Supplementary Figure 1). There were no deviation of HWE and no publication bias (Supplementary Table 4 and Supplementary Figure 3). Only ethnicity, OGTT criteria and genotyping methods had enough studies for subgroup analysis. Subgroup analysis according to ethnicity showed the risk allele was not significantly associated with GDM in Asian population or Caucasian population (Table 2). Only studies which used 100 g in OGTT for GDM diagnosis had significant association in GDM risk (pooled OR 1.88, 95% CI 1.21–2.94, p = 0.005; Table 2), while studies which used 75 g in OGTT for GDM diagnosis had no significance association (pooled OR 1.31, 95% CI 0.82–2.08, p = 0.261; Table 2). Studies used method other than Taqman assay as genotype detection (pooled OR 1.77, 95% CI 1.33–2.35, p < 0.001) were also significant associated between the risk alleles and GDM (Table 2).

*PPARG* (*rs1801282*) There were 10 included articles which studied the rs1801282 polymorphism. Meta-analysis showed that the G allele was not significantly associated with the increased risk of GDM (pooled OR 0.89, 95% CI 0.75–1.05, p = 0.154; Heterogeneity I^2^ 30.8%, p = 0.162; Table 1 and Supplementary Figure 1). HWE was significant in 2 studies^29^,^31^ (Supplementary Table 4), the significant association was not maintained when these studies with excluded (pooled OR 0.86, 95% CI 0.68–1.08, p = 0.181; Heterogeneity I^2^ 44%, p = 0.085; Table 1 and Supplementary Figure 2). Funnel plot analysis indicated potential publication bias in 1 study^32^ (Supplementary Figure 3), there was no significant association even when this study was also excluded (pooled OR 0.95, 95% CI 0.80–1.13, p0.587; Heterogeneity I^2^ 16.7%, p = 0.302; Table 1 and Supplementary Figure 4). Again only ethnicity, OGTT criteria and genotyping methods have enough studies for subgroup analysis. Subgroup analysis on ethnicity showed the risk allele was significantly associated with decreased risk of GDM in Asian populations (pooled OR 0.72, 95% CI 0.56–0.93, p = 0.011; I^2^ 24.8%, p = 0.256), but not in Caucasian populations (pooled OR 1.07, 95% CI 0.91–1.18, p = 0.611; I^2^ 0%, p = 0.474; Table 2). Studies which used either 75 g or 100 g in OGTT for GDM diagnosis had no significant association between the risk alleles and GDM (Table 2). If studies with potential publication bias were not included, the above significant association was not maintained (Supplementary Table 5). Studies used methods either Taqman assay or other assays as genotype detection were also not significant (Table 2).

### Variant involved in inflammation

*TNF-α* (*rs1800629*) There were 3 included articles which studied the rs1800629 polymorphism. Meta-analysis showed no significant association between rs1800629 and GDM risk (pooled OR 1.38, 95% CI 0.37–5.16); P = 0.633 Table 1 and Supplementary Figure 1). HWE was significant in 1 study^24^ (Supplementary Table 4), the association of rs1800629 in GDM risk become significant when this study with excluded (pooled OR 2.69, 95% CI 1.28–5.68; p = 0.009, Table 1 and Supplementary Figure 2). There was no potential publication bias (Supplementary Figure 3) and there were not enough studies for subgroup analysis.

## Discussion

Previous meta-analysis identified few genetic polymorphism associated with GDM^10^,^11^. However, these studies have certain limitations, such as single or few variants per each study, insufficient sample sizes, study heterogeneity and neglected confounding factors such as maternal age, BMI, diabetic history, etc. In our current study, we conducted a larger-scale meta-analysis and reduced the study heterogeneity to confirm the association between the risk alleles and GDM. In addition, we further carried out subgroup-analysis of ethnicity, OGTT criteria, genotyping methods and sample size in order to obtain more in-depth interpretation. Our meta-analysis included the alleles in *IGF2BP2*, *MTNR1B*, *TCF7L2*, *IRS1*, *TNF-α*, and *PPARG* genes, and showed the risk alleles of *MTNR1B*, *TCF7L2* and *IRS1*, but not *PPARG*, were significantly associated with increased risk of GDM with or without study heterogeneity, HWE derivation and publication bias. The results confirmed previous meta-analyses that the risk allele of rs7903146 in *TCF7L2*^33^ and rs1801278 in *IRS1* and rs10830963 in *MTNR1B* were associated with increased risk of GDM^34^, while there was no significant association between rs1801282 polymorphism in *PPARG* and GDM risk^35^. However, the risk alleles of *IGF2BP2* and *TNF-α* were significantly associated with increased risk of GDM only after studies which failed HWE were excluded, which had not been identified in previous studies^11^, indicating its potential association in GDM.

**Figure 2:**
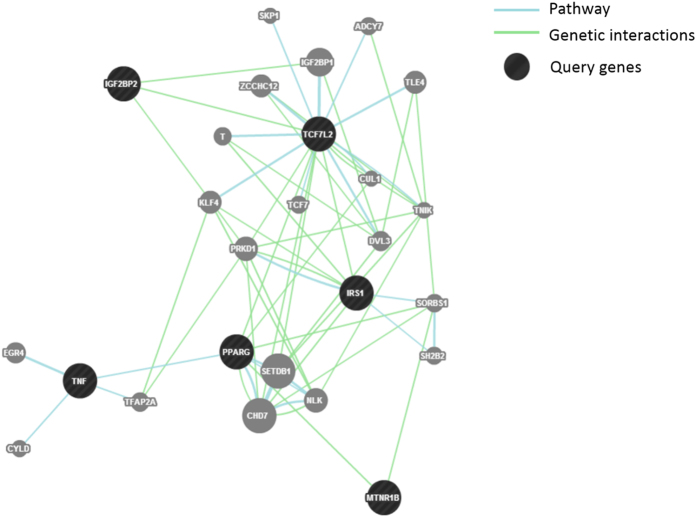
Gene pathway and genetic interactions of the SNPs in GDM.

The underlying mechanism of GDM include impaired β-cell function, and decreased insulin sensitivity Women with GDM had a higher risk of developing diabetes than those with normal blood glucose after pregnancy^36^. It has been suggested that GDM and T2DM may share common pathogenic pathways^37^. GDM is a multifactorial metabolic disorder in which loci interact with environmental factors as well as family history and obesity^38^. Some studies examined the association of genetic polymorphism between GDM and T2D and supported the hypothesis that both had common genetic background^18^,^29^,^39^. In our study, only *MTNR1B*^40^ and *TCF7L2*^41^ involved in impaired insulin secretion, and *IRS1*^42^ involved in insulin resistance in T2D were significantly associated with GDM. It further supports impaired insulin secretion and insulin resistance may involve in the pathogenesis of GDM as in T2D. By using Gene Interaction Network (GeneMENIA), *TNF-α* and *PPARG* showed direct link to each other under category of gene pathway. It indicates the direct gene-gene interactions between *TNF-α* and *PPARG* (Fig. 2). Lin *et al*. confirm the interactions by radiation hybrid genotyping in the mammalian genome^43^. Although the association between *TNF-α* and *PPARG* has been shown, their direct genetic interaction in GDM is still unclear. Obesity and insulin resistance are associated with inflammatory factors and play a pivotal role in the development of T2D and GDM^44^. *TNF-α*, as a pro-inflammatory cytokine, can activate some signaling pathways to inhibit the insulin activity^45^. However there are not enough studies of *TNF-α* to show the relationship between BMI and GDM. On the other hand, *TCF7L2* showed direct link with *IGF2BP2*, *IRS1* and *PPARG*, but indirectly with *MTNR1B*, indicating the potential genetic association between *TCF7L2* and other genes important for T2D and/or GDM. Gene-gene interactions would provide possibility for future genetic research.

Prevalence of GDM in Asian countries is now higher than that in other countries^6^. In our subgroup-analysis, only 2 study populations, Asian and Caucasian, could be compared. There were not enough studies included other populations for the comparison. The results showed that *TCF7L2* (rs7903146) and *PPARG* (rs1801282) were significantly associated with increased/decreased risk of GDM in Asian population, mostly from China, but not in Caucasian population. All 7 previous genetic studies shows the significant association of *TCF7L2* (rs7903146) in Caucasian populations were positive in 6 out 7 studies with highest OR as 2.04^46^, but negative in 1 study^30^ with the lowest OR as 0.69. In contrast, genetic studies shows the association in Asian populations were positive in 2 out of 3 studies with highest OR as 2.06^47^ but negative in 1 study^48^ with OR as 1.08. For all 5 previous genetic studies show the negative association of *PPARG* (rs1801282) in Caucasian populations but only 1 out of 5 studies in Asian population were positive^32^. Here our subgroup results suggest underlying ethnic predisposition of *TCF7L2* and *PPARG* in the development of GDM in Asian population only, but larger scale study will be necessary to confirm the results. In addition, larger pre-pregnancy BMI (≥25) has significant association in *MTNR1B* (rs10830963) with GDM, indicating the potential effects of obesity on the role of *MTNR1B* mutation in GDM risk^10^. Apart from ethnicity and obesity, genotype and OGTT test methods also affect the genetic association of the risk alleles and GDM. Genotyping in *TCF7L2* (rs7903146) and *IRS1* (rs1801278) were significantly associated with increased risk of GDM in other methods, including PCR-RFLP, rather than Taqman assay. PCR-RFLP is a rapid, simple and inexpensive genotyping technique that exploits variations in homologous DNA sequences but poor in conditions of compound heterozygous mutation^49^ and non-coding regions that the restriction enzymes may not work properly^50^. In our study no studied variants were compound heterozygous mutation but rs7903146 was a non-coding allele. The identified significant association may imply potential error in the mutation detection method by PCR-RFLP method, the results should be validated by Sanger sequencing, the gold standard of the genotyping. On the other hand, the OGTT test using 100 g, but not 75 g, was significantly associated with increased GDM risk in IRS-1 (rs1801278) only. One-step 75 g OGTT was recommended by The international Association of Diabetes and Pregnancy Study Groups (IADPSG) while two-step 100 g OGTT was recommended by the National Diabetes Data Group (NDDG) and Carpenter and Coustan for GDM diagnosis^5^. Compared with 100 g OGTT, 75 g OGTT would increase the prevalence of GDM twofold to threefold if using the Carpenter and Coustan’s diagnostic criteria. The 100 g OGTT might increase the sensitivity to detect the genetic polymorphism in IRS-1. IRS-1 is involved in insulin resistance, how different glucose loading and threshold of abnormal plasma glucose levels affect the insulin resistance remained to elucidate.

Although this present study is comprehensive, included more variants for meta-analysis and carried out subgroup-analysis, there were several limitations. Firstly, more accurate analysis using the adjusted data by age and BMI would be preferable to direct use of unadjusted raw data. Secondly, environmental factors including cigarette smoking, alcohol consumption, and dietary habit are important confounding factors which should preferably be considered and recorded in genetic studies. Thirdly, due to the limitation of included studies with small sample sizes, the odd ratio are small and the results will be seriously affected by any studies removed due to significant heterogeneity, HWE and publication bias. Since a strict minimal number of studies was required for meta-analysis, some of important variants of *CDKAL1*^51^, *KCNJ11*^37^and *GCK*^52^could not be included. A genome-wide association study has demonstrated that genetic variants in CDKAL1 gene and near MTNR1B are associated with GDM in Korean women (p < 5.0 × 10^−8^), however there is no other genome-wide association study available to extract the variant information for present meta-analysis. The genetic association of these variants with GDM should also be included in future if sufficient association studies become available.

Overall GDM was associated with rs10830963, rs7903146, and rs1801278, but only rs7903146 and rs1801282 were significant in Asian populations. While rs1801278 and rs7903146 were significantly affected by OGTT protocol and genotyping methods. In summary, the present study dissected the effects of subgroup information on the genetic association of GDM, which not only confirmed the genetic association for some of the variants, but also to explore the effects of ethnicity, diagnosis criteria, genotype methods and BMI on the results. These factors should be considered in the association between the genetic variants and the risk of GDM in future genetic studies.

## Additional Information

**How to cite this article**: Wu, L. *et al*. Genetic variants associated with gestational diabetes mellitus: a meta-analysis and subgroup analysis. *Sci. Rep*. **6**, 30539; doi: 10.1038/srep30539 (2016).

## Supplementary Material

Supplementary Information

## Figures and Tables

**Table 1:** Associations between genetic variants and GDM risk.

Gene	Variant	Minor allele	Exclusion	Number of studies	Sample size (cases/controls)	OR(95% CI)	P (Z)^a^	Heterogeneity	
								I^2^	P (Q)^b^
IGF2BP2	rs4402960	T	No^c^	3	1688/1712	1.12 (0.89, 1.40)	0.353	68.4%	0.042
			HWE & Funnel^e^	2	1593/1671	1.22 (1.09, 1.36)	＜0.001	0%	0.637
MTNR1B	rs10830963	G	No^c^	7	2705/4325	1.31 (1.18, 1.47)	＜0.001	44.2%	0.097
			HWE^d^	5	2548/3022	1.28 (1.14, 1.44)	＜0.001	50.3%	0.090
TCF7L2	rs7903146	T	No^c^	9	3206/6334	1.41 (1.16, 1.72)	0.001	80.8%	<0.001
			HWE^d^	7	2127/2871	1.40 (1.03, 1.90)	0.032	85.1%	<0.001
			HWE & Funnel^e^	6	1866/2495	1.57 (1.38, 1.79)	＜0.001	9%	0.359
IRS1	rs1801278	T	No^c^	5	1307/1973	1.53 (1.08, 2.15)	0.015	49.6%	0.094
PPARG	rs1801282	G	No^c^	10	2929/6969	0.89 (0.75, 1.05)	0.154	30.8%	0.162
			HWE^d^	8	2544/3015	0.86 (0.68, 1.08)	0.181	44.0%	0.085
			HWE & Funnel^e^	7	2365/2835	0.95 (0.80, 1.13)	0.587	16.7%	0.302
TNF-α	rs1800629	A	No^c^	3	196/181	1.38 (0.37, 5.16)	0.633	85.4%	0.001
			HWE^d^	2	86/79	2.69 (1.28, 5.68)	0.009	24.5%	0.25

^a^P value for Z test.

^b^P value for the Cochran χ^2^ Q test.

^c^No exclusion of studies for meta-analysis.

^d^Studies after removing articles deviated from Hardy-Weinberg equilibrium (HWE) in control (P < 0.05) for meta-analysis.

^e^Studies after removing articles deviated from Hardy-Weinberg equilibrium in control (P < 0.05) and outliers in funnel plot (Funnel) for meta-analysis.

**Table 2:** Subgroup analysis of genetic variants in GDM (with HWE excluded only).

Subgroup	Number of studies	Number of cases	Number of controls	OR(95% CI)	P (Z)^a^	Heterogeneity	
						I^2^	P (Q)^b^
**MTNR1B rs10830963**							
Ethnicity							
Asian	4	2090	2600	1.23 (1.10, 1.38)	<0.001	37.7%	0.186
Caucasian	1	458	422	1.49 (1.22, 1.82)	<0.001	NA	NA
OGTT							
75 g	3	895	993	1.38 (1.20, 1.57)	<0.001	0.0%	0.375
100 g	2	1653	2029	1.20 (1.01, 1.44)	0.043	72.6%	0.056
Genotype method							
Taqman assay	3	2111	2451	1.28 (1.08, 1.51)	0.004	72.2%	0.027
Others	2	437	571	1.29 (1.08, 1.54)	0.005	0.0%	0.375
Sample size							
small	1	87	91	1.53 (1.01, 2.32)	0.047	NA	NA
large	4	2461	2931	1.27 (1.12, 1.44)	<0.001	58.4%	0.066
Pre-BMI							
<25	2	812	1130	1.22 (0.9, 1.65)	0.202	54.2%	0.14
≥25	1	350	480	1.24 (1.02, 1.51)	0.033	NA	NA
**TCF7L2 rs7903146**							
Ethnicity							
Asian	3	1008	801	1.58 (1.12, 2.23)	0.009	39.1%	0.194
Caucasian	4	1119	2070	1.32 (0.86, 2.03)	0.212	91.4%	0.212
OGTT							
75 g	5	1219	2170	1.43 (0.97, 2.09)	0.07	89.7%	<0.001
100 g	2	908	701	1.36 (0.94, 1.97)	0.100	12.3%	0.286
Genotype method							
Taqman assay	4	1839	2590	1.24 (0.82, 1.87)	0.306	90.1%	<0.001
Others	3	288	281	1.73 (1.20, 2.50)	0.003	49.8%	0.136
**IRS1 rs1801278**							
Ethnicity							
Asian	2	262	400	2.15 (0.80, 5.74)	0.128	50.3%	0.156
Caucasian	3	1045	1573	1.41 (0.99, 2.01)	0.059	56.3%	0.101
OGTT							
75 g	2	736	1296	1.31 (0.82, 2.08)	0.261	70.3%	0.067
100 g	3	571	677	1.88 (1.21, 2.94)	0.005	6.8%	0.342
Genotype method							
Taqman assay	1	588	1189	1.04 (0.75, 1.45)	0.805	NA	NA
Others	4	719	784	1.77 (1.33, 2.35)	<0.001	0.0%	0.515
**PPARG rs1801282**							
Ethnicity							
Asian	5	1259	1126	0.72 (0.56, 0.93)	0.011	24.8%	0.256
Caucasian	3	1285	1889	1.07 (0.91, 1.18)	0.611	0.0%	0.474
OGTT							
75 g	5	1519	2242	0.98 (0.86, 1.12)	0.812	51.7%	0.082
100 g	3	1025	773	0.80 (0.53, 1.21)	0.294	24.3%	0.267
Genotype method							
Taqman assay	2	1505	1864	0.95 (0.71, 1.26)	0.706	55.9%	0.132
Others	6	1039	1151	0.87 (0.71, 1.05)	0.141	45.3%	0.104

^a^P values in subgroups.

^b^P values for Cochran’s Q statistic test used to assess the heterogeneity.

NA not available.
