# Supplementary material for: Genetic variants associated with gestational diabetes mellitus: a meta-analysis and subgroup analysis
Source: Sci Rep. 2016 Jul 29;6:30539. doi: 10.1038/srep30539 (PMC4965817; doi:10.1038/srep30539)
Supplement: Supplementary Information [file srep30539-s1.pdf]

Genetic variants associated with gestational diabetes mellitus: a meta-analysis and subgroup analysis

Ling Wu, Long Cui, Wing Hung Tam, Ronald C.W. Ma & Chi Chiu Wang

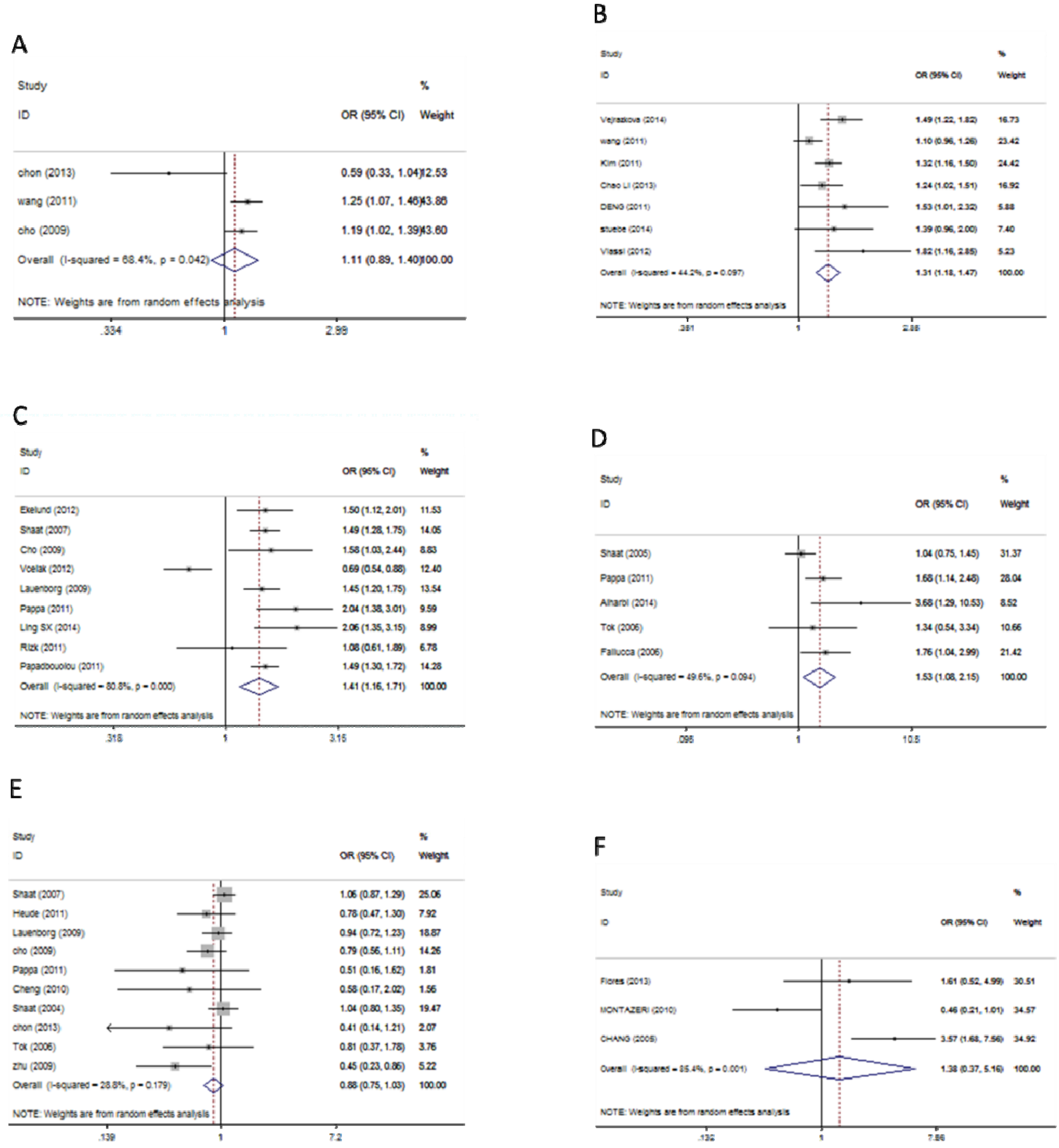

Supplementary Figure 1 Forest plots of genetic variants in GDM with all included studies. (A) IGF2BP2 rs4402960, (B) MTNR1B rs10830963, (C) TCF7L2 rs7903146, (D) IRS1 rs1801278, (E) PPARG rs1801282, (F) TNF-a rs1800629. The shadowed squares and their lateral tips indicate the ORs and the corresponding 95% CIs in individual studies, The central lines and lateral tips of the diamonds indicate the pooled ORs and the corresponding 95% CIs. The solid vertical lines indicate no effect.

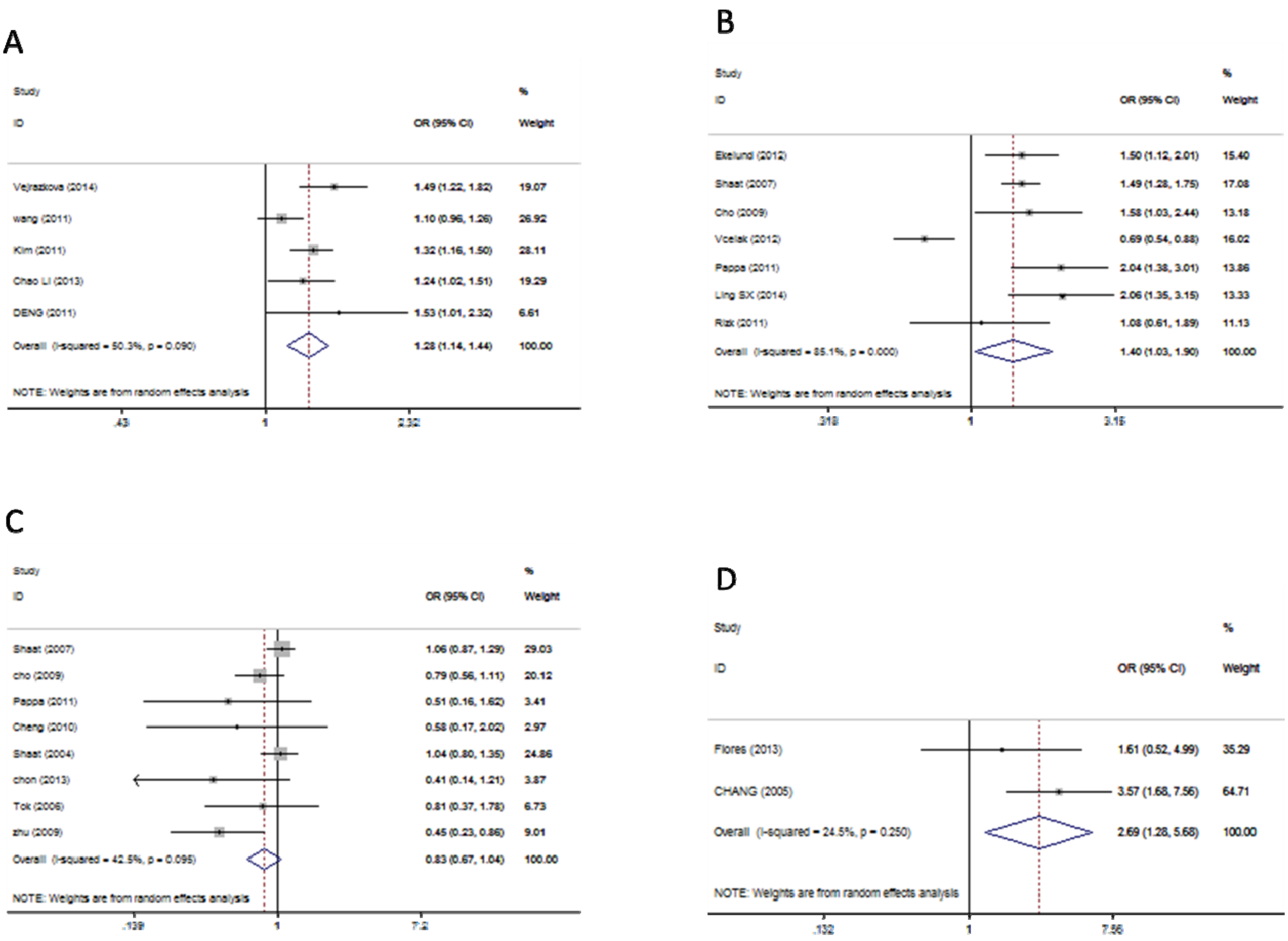

Supplementary figure 2 Forest plots of genetic variants in GDM with studies after removing articles deviated from Hardy-Weinberg equilibrium in control (P<0.05). (A) MTNR1B rs10830963, (B) TCF7L2 rs7903146, (C) PPARG rs1801282, (D) TNF- α rs1800629. The shadowed squares and their lateral Tips indicate the ORs and the corresponding 95% CIs in individual studies, The central lines and lateral tips of the diamonds indicate the pooled ORs and the corresponding 95% CIs. The solid vertical lines indicate no effect.

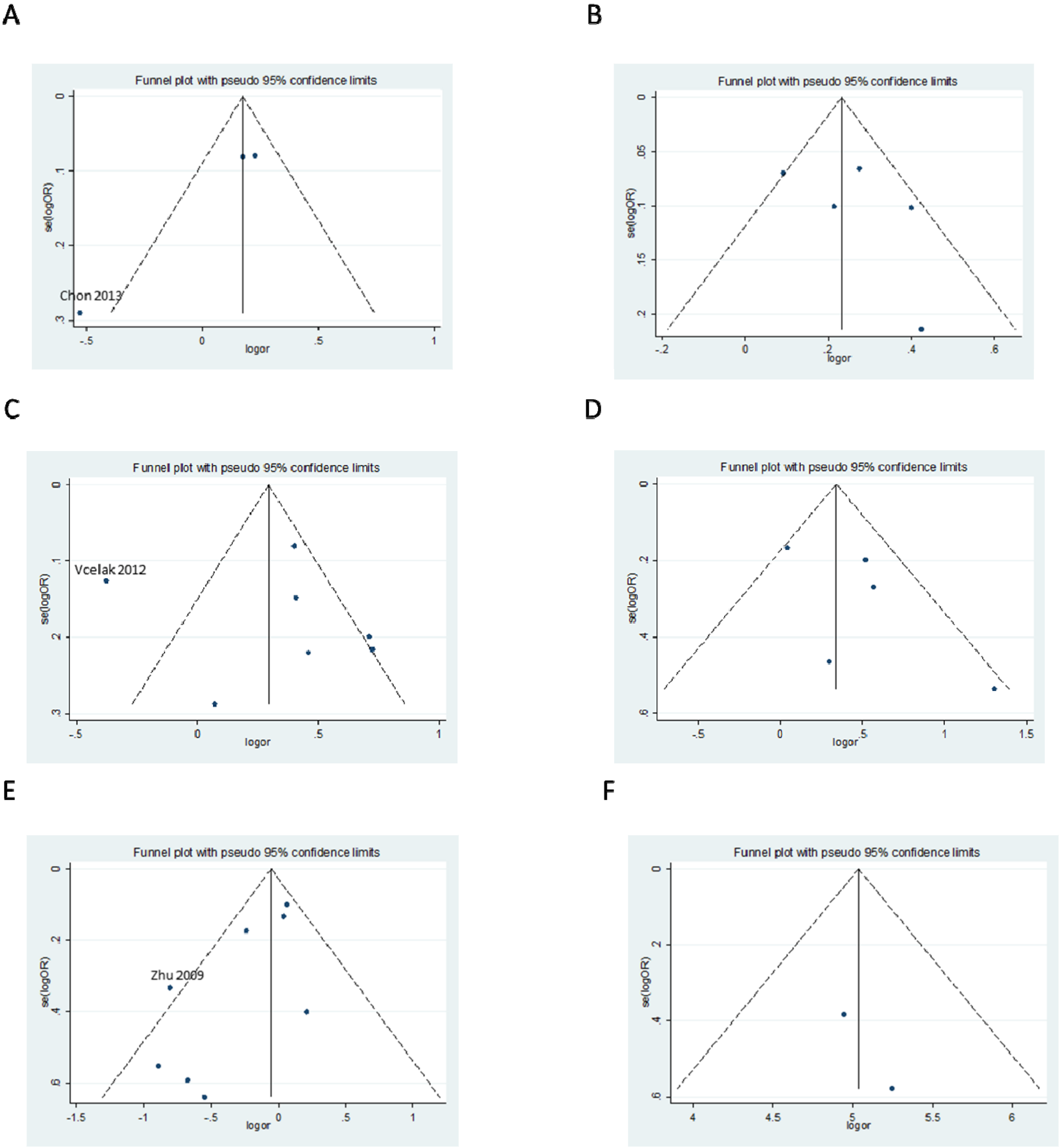

Supplementary figure 3 Funnel plots on the association of allelic models for GDM risk in a random-effects model (Egger's test). (A) IGF2BP2 rs4402960, (B) MTNR1B rs10830963, (C) TCF7L2 rs7903146, (D) IRS1 rs1801278, (E) PPARG rs1801282, (F) TNF-alpha rs1800629. Se(logOR) as the vertical axis and logOR as horizontal axis.

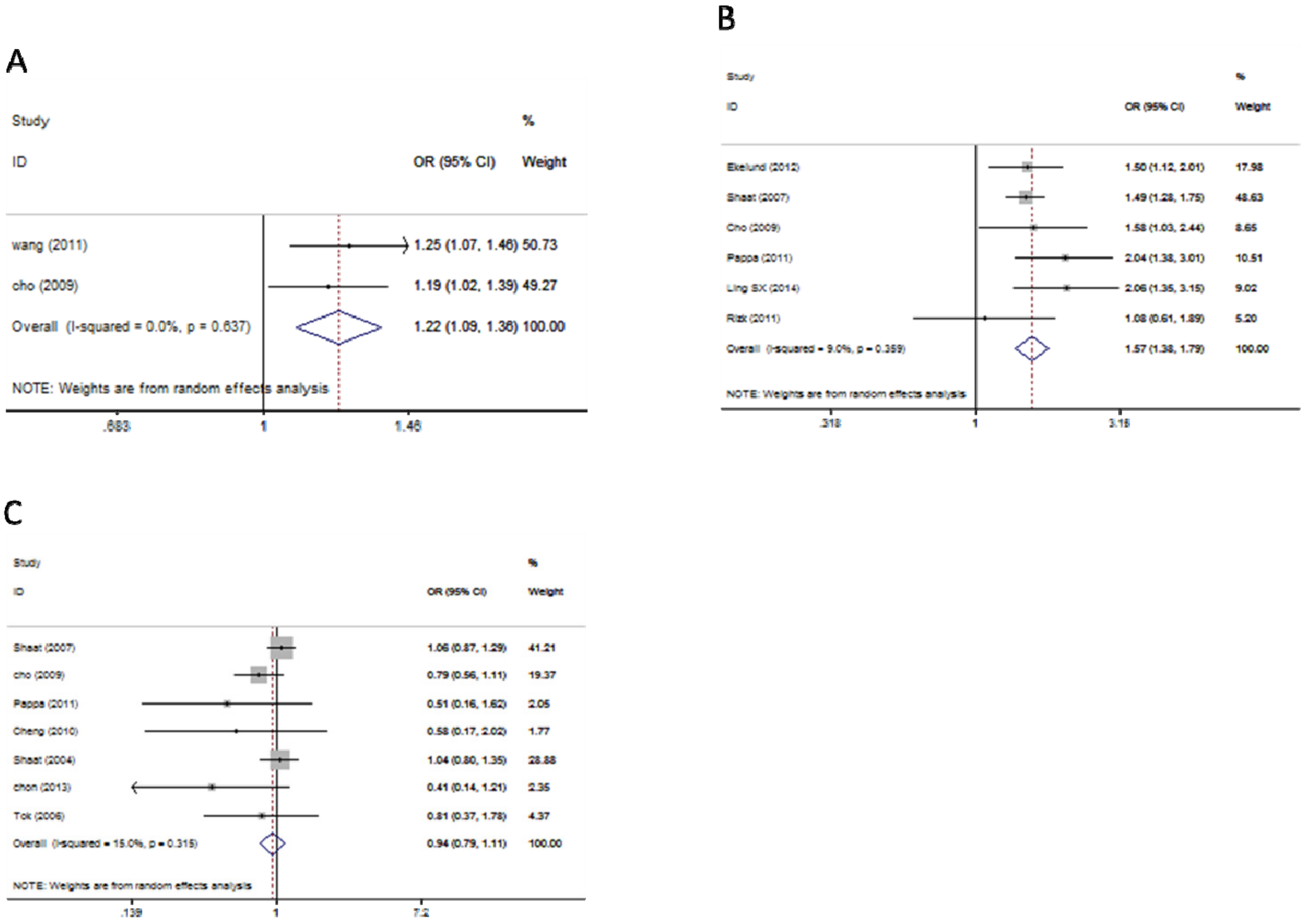

Supplementary figure 4 Forest plots of genetic variants in GDM with studies after removing articles deviated from both Hardy-Weinberg equilibrium in control (P<0.05) and publication bias (funnel plot outliers) together or only publication bias (funnel plot outliers) alone. (A) IGF2BP2 rs4402960, (B) TCF7L2 rs7903146, (C) PPARG rs1801282. The shadowed squares and their lateral tips indicate the ORs and the corresponding 95% CIs in individual studies, the central lines and lateral tips of the diamonds indicate the pooled ORs and the corresponding 95% CIs. The solid vertical lines indicate no effect.

Genetic variants associated with gestational diabetes mellitus: a meta-analysis and subgroup analysis

Ling Wu, Long Cui, Wing Hung Tam, Ronald C.W. Ma & Chi Chiu Wang

Supplementary Figure 5: Chromosome locations of genes and SNPs

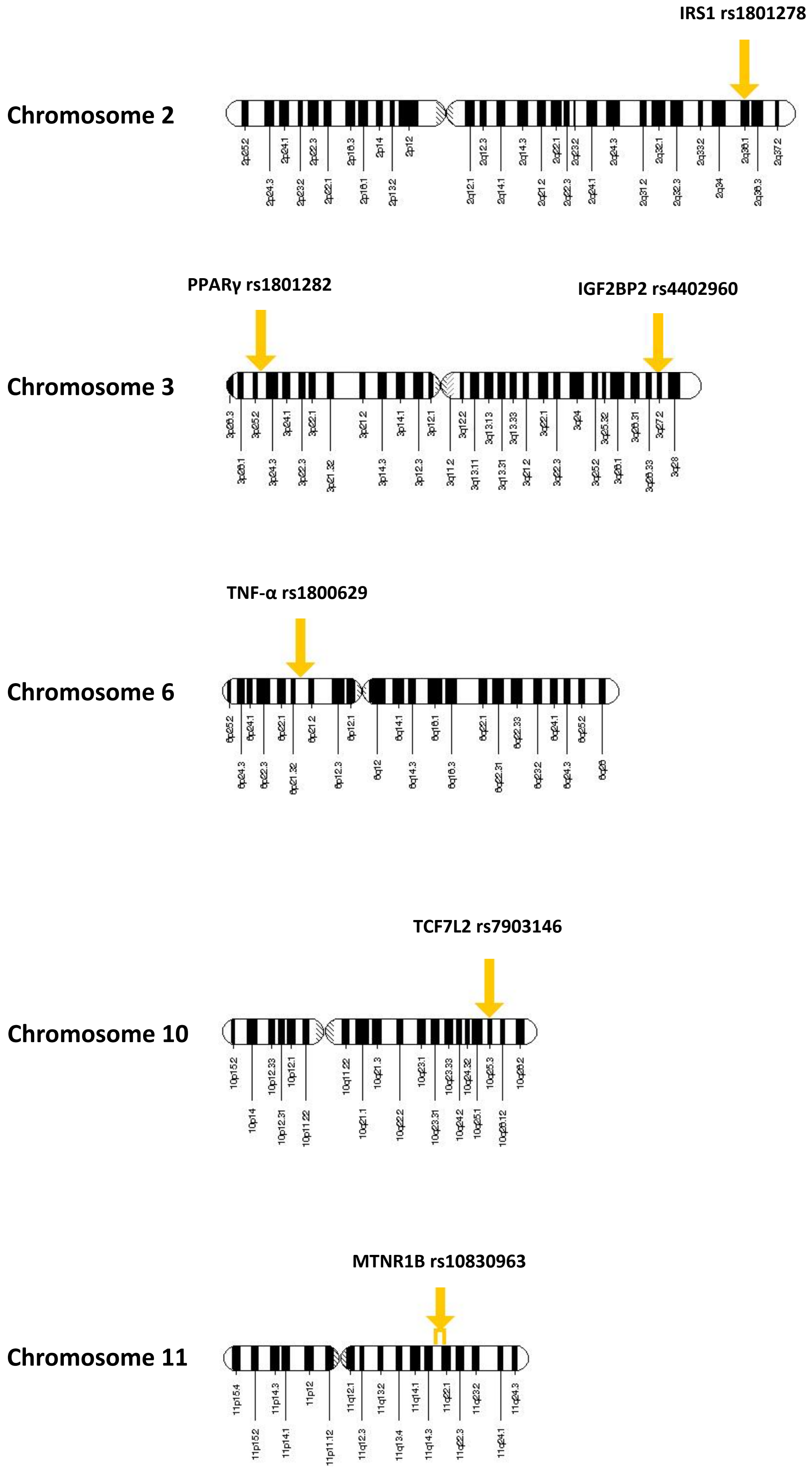

Genetic variants associated with gestational diabetes mellitus: a meta-analysis and subgroup analysis

Ling Wu, Long Cui, Wing Hung Tam, Ronald C.W. Ma & Chi Chiu Wang

Supplementary Table 1: Sample sizes estimate (OSSE) of the genetic variants on GDM

Gene and SNP	80% CI	90% CI	95% CI
MTNR1B rs10830963	336	449	556
TCF7L2 rs7903146	20,278	27,151	33,580
IRS1 rs10830963	777	1,040	12,86
PPARG rs1801282	2,071	2,772	3,428

Supplementary Table 2: Details of genes and genetic variants in GDM

Gene name	Symbol	Chromosome location	Variants	Gene function
Insulin-like growth factor 2 mRNA-binding protein 2	IGF2BP2	3q27.2	rs4402960	Insulin secretion
Melatonin receptor 1B	MTNR1B	11q21-q22	rs10830963	Insulin secretion
Transcription factor 7-like 2	TCF7L2	10q25.3	rs7903146	Insulin secretion
Insulin receptor substrate 1	IRS1	2q36	rs1801278(Gly972Arg)	Insulin resistance
Peroxisome proliferator-activated receptor gamma	PPARG	3p25	rs1801282(Pro12Ala)	Insulin resistance
Tumor necrosis factor alpha	TNF-α	6p21.3	rs1800629(-308G/A)	Inflammation

Supplementary Table 3: Characteristics of the included genetic association studies of GDM

Studies	Year	Country	Sample size		Diagnosis criteria[a]		Mean age	Mean BMI[b]	Mean Pre-BMI[c]	Genotype method[d]
			Cases	Controls	Cases(mmol/L)	Controls(mmol/L)	Cases/Controls	Cases/Controls	Cases/Controls	
**IGF2BP2 rs4402960**										
Chon	2013	Korea	95	41	2012 ADA	50g 1h<7.8	34.3/32.6	29.2/26.8	25.7/22	PCR
Wang	2011	China	725	1039	2012 ADA	50g 1h<7.8	32/30	NA/NA	21.7/21.5	Taqman
Cho	2009	Korea	868	632	NDDG	0h<6.1 HbA_{1C}<5.8%	32/64.7	23.1/23.3	NA/NA	Taqman
**MTNR1B rs10830963**										
Stuebe	2014	Carolina	80	1205	2012 ADA	50g 1h<7.8	NA/NA	NA/NA	NA/NA	NA
Vejrazkova	2014	Czech	458	422	1999 WHO	0h<5.6	34.1/34.8	24.3/23.7	NA/NA	Taqman
Chao	2013	China	350	480	IADPSG	Normal glucose	32.4/32.0	NA/NA	25.3/24.7	PCR-RFLP
Vlassi	2012	Greece	77	98	2012 ADA	50g 1h<7.8	35.5/31.4	NA/NA	25.8/26.8	PCR-RFLP
DENG	2011	China	87	91	5.6-10.3-8.6	Normal glucose	31.8/29.7	NA/NA	23.6/21.5	Sequencing
Wang	2011	China	725	1039	2012 ADA	50g 1h<7.8	32/30	NA/NA	21.7/21.5	Taqman
Kim	2011	Korea	928	990	2012 ADA	50g 1h<7.8	33.1/32.2	23.3/21.4	NA/NA	Taqman
**TCF7L2 rs7903146**										
Ling	2014	China	100	100	IADPSG	50g 1h<7.8	30/29	27.4/24.2	NA/NA	PCR
Ekelund	2012	Sweden	125	476	1999 WHO	75g 2h<7.8	32.8/32.2	30.8/25.5	NA/NA	Taqman
Vcelak	2012	Czech	261	376	1999 WHO	Normal glucose	32.8/29.9	23.8/23.3	NA/NA	Taqman
Papadbouolou	2011	Sweden	803	1110	IADPSG	75g 2h<9.0	NA/NA	NA/NA	NA/NA	PCR
Pappa	2011	Greek	148	107	5.3-7.8-6.7	75g 2h<7.8	32.5/26.7	NA/NA	26/24.26	PCR-RFLP
Rizk	2011	Qatar	40	74	NA	NA	NA/NA	NA/NA	NA/NA	NA
Lauenborg	2009	Danish	276	2353	1999 WHO	Normal glucose	43.1/45.2	28.9/25	NA/NA	Taqman
Cho	2009	Korean	868	627	NDDG	0h<6.1 HbA_{1C}<5.8%	32/64.7	23.1/23.3	NA/NA	Taqman
Shaat	2007	Sweden	585	1111	2h ≥9.0	75g 2h<9.0	32.3/30.5	NA/NA	NA/NA	Taqman
**IRS1 rs1801278**										
Alharbi	2014	Saudi	200	300	2012 ADA	50g 1h<7.8	32.4/31.3	34.4/33.3	NA/NA	PCR-RFLP
Pappa	2011	Greece	148	107	5.3-7.8-6.7	Normal glucose	32.5/26.6	NA/NA	26/24	PCR-RFLP
Tok	2006	Turkey	62	100	NDDG	50g 1h<7.8	33.6/NA	NA/NA	26.5/NA	PCR-RFLP
Fallucca	2006	Italy	309	277	2012 ADA	50g 1h<7.8	34.1/32.7	NA/NA	25.4/23.8	PCR-RFLP
Shaat	2005	sweden	588	1189	2h ≥9.0	2h<9.0	32.2/30.5	24.5/23.1	NA/NA	Taqman
**PPARG rs1801282**										
Chon	2013	Korea	95	41	2012 ADA	50g 1h<7.8	34.3/32.6	29.2/26.8	25.7/22	PCR
Heude	2011	France	109	1571	50g≥7.8	50g 1h<7.8	NA/NA	NA/NA	26.8/23.2	Taqman
Pappa	2011	Greece	148	107	5.3-7.8-6.7	75g 2h<7.8	32.5/26.7	NA/NA	26/24.3	RFLP
Cheng	2010	China	55	173	5.6-10.3-8.6	50g 1h<7.8	27/29.6	NA/NA	NA/NA	RFLP
Cho	2009	Korea	868	632	NDDG	0h<6.1 HbA_{1C}<5.8%	32/64.7	23.1/23.3	NA/NA	Taqman
Zhu and Wu	2009	China	179	180	5.6-10.3-8.6	Normal glucose	28.1/27.4	24.6/23.4	NA/NA	RFLP
Lauenborg	2009	Denmark	276	2383	1999 WHO	Normal glucose	43.1/45.2	28.9/25	NA/NA	Taqman
Shaat	2007	Sweden	637	1232	2h ≥9.0	75g 2h<9.0	32.3/30.5	NA/NA	NA/NA	Taqman
Tok	2006	Turkey	62	100	NDDG	50g 1h<7.8	33.5/31.6	NA/NA	26.6/23	RFLP
Shaat	2004	Sweden	500	550	2h ≥9.0	75g 2h<9.0	32.3/NA	30.1/NA	NA/NA	RFLP
**TNF-α rs1800629**										
Flores	2013	Mexico	51	44	IADPSG	Normal glucose	33/25.6	29.6/24.5	NA/NA	PCR-RFLP
Montazeri	2010	Malaysia	110	102	1999 WHO	50g 1h<7.8	NA/NA	NA/NA	NA/NA	PCR-RFLP
Chang	2005	China	35	35	5.6-10.5-9.2	Normal glucose	30/28	NA/NA	NA/NA	PCR-RFLP

[a]IADPSG, the International Association of Diabetes and Pregnancy Study Groups; ADA, American Diabetes Association; NDDG, National Diabetes Data Group; WHO, World Health Organization; 2h; postprandial blood sugar 2 hours; 50g, 50 gram oral glucose tolerance test

[b]BMI, Body Mass Index;

[c]Pre-BMI, Pre-pregnancy Body Mass Index

PCR, polymerase chain reaction; RFLP, restriction fragment length polymorphism

NA, not available

Genetic variants associated with gestational diabetes mellitus: a meta-analysis and subgroup analysis
Ling Wu, Long Cui, Wing Hung Tam, Ronald C.W. Ma & Chi Chiu Wang

Supplementary Table 4: Distribution of genotypes and alleles in individual studies

Studies	Cases					Controls					HWE
	Genotypes			alleles		genotypes			alleles		(p value)
**IGF2BP2 rs4402960 (G/T)**	GG	GT	TT	G	T	GG	GT	TT	G	T	
Chon	57	30	7	144	44	15	24	2	54	28	0.053
Wang	371	278	56	1020	390	605	361	59	1571	479	0.596
Cho	389	365	103	1143	571	313	257	57	883	371	0.685
**MTNR1B rs10830963 (C/G)**	CC	GG	GC	C	G	CC	GG	GC	C	G	
Stuebe	43	7	29	115	43	763	78	349	1875	505	<0.001
Vejrazkova	169	62	227	565	351	206	32	184	596	248	0.298
Chao	113	79	158	384	316	172	75	233	577	383	0.790
Vlassi	30	16	31	91	63	56	12	30	142	54	0.021
DENG	23	26	38	84	90	31	15	45	107	75	0.845
Wang	199	137	364	762	638	329	191	509	1167	891	0.812
Kim	217	256	435	869	947	294	203	469	1057	875	0.528
**TCF7L2 rs7903146 (C/T)**	CC	CT	TT	C	T	CC	CT	TT	C	T	
Ling SX	40	36	24	116	84	55	38	7	148	52	0.901
Ekelund	49	56	20	154	96	239	195	42	673	279	0.805
Vcelak	142	102	17	386	136	156	185	35	497	255	0.058
Papadbouolou	363	352	88	1078	528	644	384	82	1672	548	0.020
Pappa	49	81	18	179	117	62	38	7	162	52	0.720
Rizk	16	18	6	50	30	29	37	8	95	53	0.451
Lauenborg	118	125	33	361	191	1292	863	198	3447	1259	0.002
Cho	803	63	2	1669	67	596	31	0	1223	31	0.526
Shaat	271	255	59	797	373	650	392	69	1692	530	0.339
**IRS1 rs1801278 (C/T)**	CC	TT	TC	C	T	CC	TT	TC	C	T	
Alharbi	189	1	10	388	12	295	0	5	595	5	0.884
Pappa	58	17	73	189	107	60	7	40	160	54	0.924
Tok	53	0	9	115	9	89	0	11	189	11	0.561
Fallucca	271	4	34	576	42	255	0	22	532	22	0.491
Shaat	534	4	49	1117	57	1078	0	111	2267	111	0.091
**PPARG rs1801282 (C/G)**	CC	CG	GG	C	G	CC	CG	GG	C	G	
Chon	89	5	1	183	7	34	7	0	75	7	0.550
Heude	92	17	0	201	17	1265	305	1	2835	307	<0.001
Pappa	143	5	0	291	5	100	7	0	207	7	0.726
Cheng	52	3	0	107	3	157	16	0	330	16	0.524
Cho	793	71	1	1657	73	567	63	2	1197	67	0.859
Zhu and Wu	165	14	0	344	14	155	20	5	330	30	0.189
Lauenborg	201	60	4	462	68	1790	542	51	4122	644	0.003
Shaat	468	158	11	1094	180	918	298	16	2134	330	0.134
Tok	50	12	0	112	12	84	16	0	184	16	0.385
Shaat	377	120	3	874	126	423	120	7	966	134	0.643
**TNF-α rs1800629 (G/A)**	GG	GA	AA	G	A	GG	GA	AA	G	A	
Flores	43	7	1	93	9	39	5	0	83	5	0.689
Montazeri	103	4	3	210	10	94	6	2	194	20	<0.001
Chang	10	17	8	37	33	22	12	1	56	14	0.672

HWE, Hardy-Weinberg equilibrium in control

Genetic variants associated with gestational diabetes mellitus: a meta-analysis and subgroup analysis
Ling Wu, Long Cui, Wing Hung Tam, Ronald C.W. Ma & Chi Chiu Wang

Supplementary Table 5: Subgroup information of the genetic variants in GDM (with HWE and funnel outliers excluded)

Subgroup	Number of studies	Number of cases	Number of controls	OR(95%CI)	P (Z)[a]	Heterogeneity I^2	Heterogeneity P (Q)[b]
**MTNR1B rs10830963**							
Ethnicity							
Asian	4	2090	2600	1.23 (1.10,1.38)	<0.001	37.7%	0.186
Caucasian	1	458	422	1.49 (1.22,1.82)	<0.001	NA	NA
OGTT							
75g	3	895	993	1.38 (1.20,1.57)	<0.001	0.0%	0.375
100g	2	1653	2029	1.20 (1.01,1.44)	0.043	72.6%	0.056
Genotype method							
Taqman	3	2111	2451	1.28 (1.08,1.51)	0.004	72.2%	0.027
Others	2	437	571	1.29 (1.08,1.54)	0.005	0.0%	0.375
Sample size							
small	1	87	91	1.53 (1.01,2.32)	0.047	NA	NA
large	4	2461	2931	1.27 (1.12,1.44)	<0.001	58.4%	0.066
Pre-BMI							
<25	2	812	1130	1.22 (0.9,1.65)	0.202	54.2%	0.14
≥25	1	350	480	1.24 (1.02,1.51)	0.033	NA	NA
**TCF7L2 rs7903146**							
Ethnicity							
Asian	3	1008	801	1.58 (1.12,2.23)	0.009	39.1%	0.194
Caucasian	3	858	1694	1.55 (1.35,1.79)	<0.001	6.3%	0.344
OGTT							
75g	4	958	1794	1.62 (1.39,1.89)	<0.001	19.8%	0.291
100g	2	908	701	1.36 (0.94,1.97)	0.100	12.3%	0.286
Genotype method							
Taqman	3	1578	2214	1.50 (1.32,1.72)	<0.001	0.0%	0.970
Others	3	288	281	1.73 (1.20,2.50)	0.003	49.8%	0.136
**IRS1 rs1801278**							
Ethnicity							
Asian	2	262	400	2.15 (0.80,5.74)	0.128	50.3%	0.156
Caucasian	3	1045	1573	1.41 (0.99,2.01)	0.059	56.3%	0.101
OGTT							
75g	2	736	1296	1.31 (0.82,2.08)	0.261	70.3%	0.067
100g	3	571	677	1.88 (1.21,2.94)	0.005	6.8%	0.342
Genotype method							
Taqman assay	1	588	1189	1.04 (0.75,1.45)	0.805	NA	NA
Others	4	719	784	1.77 (1.33,2.35)	<0.001	0.0%	0.515
**PPARG rs1801282**							
Ethnicity							
Asian	4	1080	946	0.79 (0.59,1.05)	0.104	0.0%	0.410
Caucasian	3	1285	1889	1.04 (0.89,1.22)	0.608	0.0%	0.469
OGTT							
75g	4	1340	2062	1.03 (0.89,1.20)	0.687	0.0%	0.503
100g	3	1025	773	0.80 (0.53,1.21)	0.294	24.3%	0.267
Genotype method							
Taqman	2	1505	1864	0.95 (0.71,1.26)	0.706	55.9%	0.132
Others	5	860	971	0.85 (0.62,1.17)	0.328	13.4%	0.329

[a] p values in subgroups
[b] p value for Cochran's Q statistic test used to assess the heterogeneity
NA not available.
